# Immune Checkpoint Inhibitor Retreatment after Enfortumab Vedotin in Advanced Urothelial Carcinoma: A Descriptive Study with Exploratory Evaluation of Radiotherapy

**DOI:** 10.31662/jmaj.2025-0463

**Published:** 2026-02-06

**Authors:** Akinori Minato, Yoshihiro Sugita, Yui Mizushima, Shuki Watanabe, Takuo Matsukawa, Kazumasa Jojima, Rieko Kimuro, Katsuyoshi Higashijima, Yujiro Nagata, Ikko Tomisaki, Eiji Kashiwagi

**Affiliations:** 1 Department of Urology, School of Medicine, University of Occupational and Environmental Health, Kitakyushu, Japan

**Keywords:** immune checkpoint inhibitor, retreatment, rechallenge, urothelial carcinoma, avelumab, pembrolizumab, nivolumab, enfortumab vedotin

## Introduction

The advent of immune checkpoint inhibitors (ICIs) has revolutionized therapeutic strategies for advanced urothelial carcinoma (UC). The KEYNOTE-045, JAVELIN Bladder 100, and CheckMate 274 clinical trials have demonstrated the utility of ICIs in various disease settings ^(1)^; however, despite prior ICI exposure, most patients eventually experience disease progression. Therefore, enfortumab vedotin (EV) has become an essential treatment option, with the EV-301 trial confirming its efficacy in patients previously treated with platinum-based chemotherapy and ICIs ^(2)^. Favorable outcomes have been observed for sequential strategies involving EV in real-world practice ^(3), (4)^.

Although the THOR trial showed the efficacy of the pan-fibroblast growth factor receptor (FGFR) inhibitor erdafitinib in patients with FGFR alterations ^(5)^, the availability of targeted therapies for routine practice is limited. Consequently, ICI retreatment is considered after EV failure, particularly when no alternative therapies are available; however, prospective evidence supporting this approach for advanced UC treatment is lacking ^(6)^.

ICI retreatment is generally administered in late-line settings, when patients experience rapidly progressive disease and a high tumor burden ^(7)^. Under these circumstances, ICI retreatment may be combined with consolidative or palliative radiotherapy to achieve local control.

In this study, we retrospectively evaluated patients with advanced UC who underwent ICI retreatment to characterize treatment practices and clinical outcomes, including an exploratory analysis of patients treated with concurrent radiotherapy.

## Materials and Methods

### Study design

A retrospective review was conducted of patients with metastatic UC treated at our institution between November 2021 and May 2025. Of 56 patients who received EV monotherapy following platinum-based chemotherapy and ICI therapy, those who subsequently underwent ICI retreatment were identified. This retrospective study protocol obtained approval from the University of Occupational and Environmental Health Institutional Review Board (approval no.: UOEH21-074) and conformed to the principles of the Declaration of Helsinki. Informed consent was obtained through an opt-out process due to the retrospective study.

### Treatment and definitions

Immune checkpoint inhibitor retreatment was categorized as rechallenge and switch treatment. Rechallenge was defined as the re-administration of the same ICI class, such as an anti-programmed death 1 (PD-1) antibody, following anti-PD-1. Switch treatment was considered a change between ICI classes, such as from anti-programmed death-ligand 1 (PD-L1) to anti-PD-1 antibody. Radiotherapy was simultaneously administered with ICI retreatment to target either the main progressive lesion or symptomatic sites to achieve local control.

### Assessments

Tumor response was assessed according to the Response Evaluation Criteria in Solid Tumors version 1.1. Irradiated lesions were excluded from the evaluation. Local control was defined as the absence of progression within irradiated lesions, as determined by computed tomography. Progression-free survival (PFS) was measured from the initiation of ICI retreatment until radiographic or clinical progression or death from any cause. Local control duration (LCD) was defined as the interval from the initiation of ICI retreatment to progression within the irradiated sites. Overall survival (OS) was measured from ICI retreatment initiation to death from any cause.

### Statistical analysis

Statistical analyses were performed using EZR version 1.55 software (Saitama Medical Center, Jichi Medical University, Japan), which is a graphical user interface for R (The R Foundation for Statistical Computing, Vienna, Austria). Survival outcomes, including PFS, LCD, and OS, were estimated by the Kaplan-Meier method.

## Results

**Figure 1.**
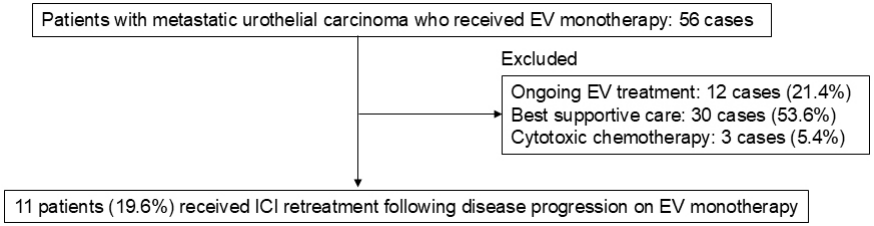
Patient flow diagram. EV, enfortumab vedotin; ICI, immune checkpoint inhibitor.

Of 56 patients with metastatic UC who received EV, 12 (21.4%) remained on EV treatment, 30 (53.6%) received best supportive care, and 3 (5.4%) received cytotoxic chemotherapy. Finally, 11 patients (19.6%) underwent ICI retreatment following disease progression on EV (Figure 1). The baseline characteristics are listed in Table 1. The median age was 72 years, and most patients were male. In most cases, the bladder was the primary tumor site. The first ICI was an anti-PD-1 antibody administered to four patients and an anti-PD-L1 antibody in seven patients. Accordingly, ICI retreatment was considered a rechallenge in four cases (36.4%) and a switch treatment in seven (63.6%). Concurrent radiotherapy was administered in nine patients (81.8%).

**Table 1.** Baseline Characteristics at the Initiation of ICI Retreatment.

Characteristic	N = 11
Age, median (range)	72 (61-75)
Sex, n (%)	
Male	9 (81.8)
Female	2 (18.2)
ECOG PS score, n (%)	
0	6 (54.5)
1	5 (45.5)
Primary tumor site, n (%)	
Upper urinary tract	4 (36.4)
Bladder	7 (63.6)
Removal of primary site, n (%)	
Yes	8 (72.7)
1st ICI treatment, n (%)	
Anti-PD-1 antibody	4 (36.4)
Anti-PD-L1 antibody	7 (63.6)
Metastatic lesion, *n* (%)	
Lymph node	9 (81.8)
Lung	4 (36.4)
Liver	2 (18.2)
Bone	4 (36.4)
Therapy lines of ICI retreatment, n (%)	
4	9 (81.8)
5	1 (9.1)
6	1 (9.1)
Type of ICI retreatment, n (%)	
Rechallenge	4 (36.4)
Switch treatment	7 (63.6)
Concurrent radiation therapy, n (%)	9 (81.8)

ECOG PS: Eastern Cooperative Oncology Group performance status; ICI: immune checkpoint inhibitor; PD-1: programmed cell death protein 1; PD-L1: programmed death-ligand 1.

Table 2 shows the treatment details and outcomes for the 11 patients who received ICI retreatment. All patients had achieved disease control during prior EV therapy before undergoing ICI retreatment. All patients subsequently received pembrolizumab as the second ICI. No objective responses were observed; however, disease control was achieved in four patients (36.4%). Notably, all four patients who experienced disease control during ICI retreatment had previously responded to their first ICI, and the target lesions at retreatment were confined to lymph node metastases. Among the four patients who achieved disease control during ICI retreatment, the PFS during their first ICI therapy was 9.9, 28.1, 2.3, and 10.1 months, respectively. The local control rate for patients receiving concurrent radiotherapy was 88.9%. With respect to biomarkers, genomic testing was conducted in five patients, none of whom were microsatellite instability-high, and one harbored an FGFR3 alteration.

**Table 2.** Treatment Summaries for Patients Who Underwent ICI Retreatment.

Case	Primary site	Radical Surgery	1st ICI	Best Response	PFS (months)	Reason for Discontinuing 1st ICI	Best response To EV	PFS (months)	2nd ICI	Target lesion	Best response To 2nd ICI	PFS (months)	Irradiated lesion	Local Control	MSI-high	FGFR3 Mutation
1	UUT	Yes	Pembrolizumab	PR	9.9	Progression	SD	4.9	Pembrolizumab	Lymph node	SD	9.8	Lymph node	Yes	NA	NA
2	Bladder	Yes	Pembrolizumab	PR	28.1	Progression	CR	10.7	Pembrolizumab	Lymph node	SD	7.0	Lymph node	Yes	NA	NA
3	UUT	No	Pembrolizumab	PD	2.5	Progression	PR	6.7	Pembrolizumab	Primary site, Lymph node, Liver, Bone, Peritoneum	PD	2.1	Liver	No	NA	NA
4	UUT	Yes	Nivolumab	PD	3.0	Progression	PR	6.0	Pembrolizumab	Lymph node, Lung	PD	1.1	Lymph node, Lung	Yes	Negative	Negative
5	Bladder	No	Avelumab	PD	1.2	Progression	SD	3.9	Pembrolizumab	Lymph node, Lung	PD	0.3	Lymph node, Lung	Yes	NA	NA
6	Bladder	Yes	Avelumab	SD	2.3	Adverse event	PR	4.4	Pembrolizumab	Lymph node	SD	4.2	None	None	NA	NA
7	UUT	Yes	Avelumab	SD	4.0	Progression	PR	7.4	Pembrolizumab	Primary site, Lymph node, Lung, Bone, Adrenal gland	PD	2.4	None	None	Negative	Negative
8	Bladder	Yes	Avelumab	PD	1.9	Progression	SD	6.0	Pembrolizumab	Liver	PD	3.2	Liver	Yes	NA	NA
9	Bladder	Yes	Avelumab	SD	10.1	Progression	SD	5.0	Pembrolizumab	Lymph node	SD	16.3	Lymph node	Yes	Negative	Negative
10	Bladder	Yes	Avelumab	SD	2.9	Progression	SD	4.4	Pembrolizumab	Lymph node, Bone, Peritoneum	PD	2.1	Bone	Yes	Negative	Positive
11	Bladder	No	Avelumab	SD	5.3	Progression	SD	9.4	Pembrolizumab	Lung, Bone	PD	2.3	Bone	Yes	Negative	Negative

1st: first; 2nd: second; EV: enfortumab vedotin; FGFR: fibroblast growth factor receptor; ICI: immune checkpoint inhibitor; MSI: microsatellite instability; NA: not assessed; PD: progressive disease; PFS: progression-free survival; PR: partial response; SD: stable disease; UUT: upper urinary tract.

**Figure 2.**
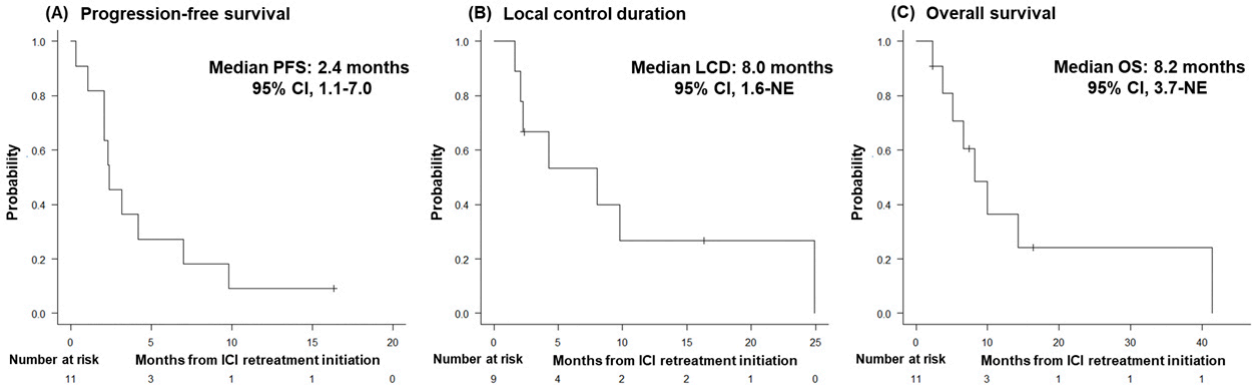
Kaplan-Meier curves showing (A) progression-free survival, (B) local control duration, and (C) overall survival in patients who were administered ICI retreatment. CI: confidence interval; ICI: immune checkpoint inhibitor; LCD: local control duration; NE: not estimable; OS: overall survival; PFS: progression-free survival.

The median follow-up time from the initiation of ICI retreatment was 7.4 months (range, 2.3-41.4). The survival outcomes are shown in Figure 2. The median PFS following ICI retreatment was 2.4 months. In contrast, the median LCD in patients who were administered concurrent radiotherapy was 8.0 months, which was comparable to the median OS of 8.2 months.

## Discussion

Previous studies evaluating ICI retreatment for UC are limited, with just one small case series ^(6)^. In the present study, all patients received ICI retreatment following disease progression on EV. To our knowledge, this is the first case series from Japan addressing ICI retreatment in UC. In our cohort, disease control was achieved during ICI retreatment exclusively in patients with lymph node-only metastases, all of whom had previously responded to their first ICI regimen. This finding is consistent with previous reports in which patients with lymph node-only metastases were more likely to derive clinical benefit from ICI therapy ^(8)^.

Studies in other malignancies, such as non-small cell lung cancer and malignant melanoma, suggest that patients who respond to their first ICI may derive benefit from retreatment ^(9), (10), (11)^. A recent meta-analysis of various cancer types (14 studies, n = 74) reported that, although the overall response rate to ICI retreatment was low, the duration of response to the first ICI may be a predictive factor ^(12)^. In that study, the pooled median PFS for ICI retreatment was 2.8 months; however, in patients whose first ICI achieved a PFS of ≥12 months, the median PFS with retreatment improved to 5.1 months ^(12)^. In our study, with the exception of one case with a short PFS of 2.3 months, the remaining patients had relatively long PFS during their first ICI therapy, suggesting a trend consistent with previous reports indicating that a durable response to initial ICI treatment may predict clinical benefit from retreatment.

Microsatellite instability-high and PD-L1 expression are established biomarkers for ICI therapy. Yearley et al. ^(13)^ found that PD-L2 was expressed in 18% of bladder cancers, including a subset that were PD-L1-negative, and PD-L2 positivity independently predicted improved outcomes with pembrolizumab. These results suggest that PD-L2 may serve as a complementary biomarker for anti-PD-1 retreatment strategies in UC, particularly in the context of switch treatment from an anti-PD-L1 antibody to an anti-PD-1 antibody, as in our cohort.

Recently, the ARON-2 study reported favorable outcomes in advanced UC patients treated with radiotherapy plus pembrolizumab ^(14)^. Radiotherapy may enhance antitumor immunity through immunogenic cell death, enhanced T-cell priming, and adaptive upregulation of PD-L1 within the tumor microenvironment, thereby synergizing with ICIs ^(15)^.

This study had several limitations. First, it was retrospective with a single institutional design and a small sample size, which inherently limited the generalizability of our results. Second, the indications and targets for radiotherapy were heterogeneous. Third, we were unable to stratify the efficacy of ICI retreatment between the rechallenge and switch treatments. Moreover, considering the small sample size and the imbalance between subgroups, we could not perform a comparative analysis of treatment response or survival between patients who received concurrent radiotherapy and those who did not.

To summarize, ICI retreatment following EV provides only a modest systemic benefit, with stabilization observed exclusively in patients with lymph node-only metastases; however, concurrent radiotherapy resulted in durable local control, which supports its role as a bridging strategy to subsequent therapies in this setting. Further studies are needed to validate these findings and refine the role of ICI retreatment, particularly in the era of novel combinations, such as EV plus pembrolizumab.

## Article Information

### Acknowledgments

The authors would like to thank Enago (www.enago.jp) for the English language editing.

### Author Contributions

Contributed to the conceptualization, acquisition of patients’ data, data curation, statistical analysis, and writing of the original draft: Akinori Minato. Contributed to the acquisition of patients’ data, data curation, reviewing, and editing: Yoshihiro Sugita, Yui Mizushima, Shuki Watanabe, Takuo Matsukawa, Kazumasa Jojima, Rieko Kimuro, Katsuyoshi Higashijima, and Yujiro Nagata. Reviewed the draft: Ikko Tomisaki. Supervised the study and approved the final version of the draft: Eiji Kashiwagi.

### Conflicts of Interest

All authors declare that no financial support was received from any organization for the submitted work. Akinori Minato declares honoraria from Merck, Astellas, MSD, Ono, Bristol-Myers Squibb, and Fuso, all unrelated to this manuscript, and consulting fees from Janssen, also unrelated to this manuscript. All other authors have declared that they have no financial relationships with any organizations that might have an interest in the submitted work.

### Approval by the Institutional Review Board (IRB)

This retrospective study protocol obtained approval from the University of Occupational and Environmental Health Institutional Review Board (approval no.: UOEH21-074) and conformed to the principles of the Declaration of Helsinki. Informed consent was obtained through an opt-out process (the hospital’s website) due to the retrospective study.
